# Insular and caudate lesions release abnormal yawning in stroke patients

**DOI:** 10.1007/s00429-013-0684-6

**Published:** 2013-12-12

**Authors:** Heinz Krestel, Christian Weisstanner, Christian W. Hess, Claudio L. Bassetti, Arto Nirkko, Roland Wiest

**Affiliations:** 1 Department of Neurology, Inselspital, Bern University Hospital, University of Bern, Freiburgstrasse 10, 3010 Bern, Switzerland; 2 Department of Pediatric Neurology, Inselspital, Bern University Hospital, University of Bern, Freiburgstrasse 10, 3010 Bern, Switzerland; 3 University Institute of Diagnostic and Interventional Neuroradiology, Inselspital, University of Bern, Bern, Switzerland

**Keywords:** Chasm, Disconnection syndrome, Neurotransmitter release, Anterior cerebral circulation, MRI

## Abstract

Abnormal yawning is an underappreciated phenomenon in patients with ischemic stroke. We aimed at identifying frequently affected core regions in the supratentorial brain of stroke patients with abnormal yawning and contributing to the anatomical network concept of yawning control. Ten patients with acute anterior circulation stroke and ≥3 yawns/15 min without obvious cause were analyzed. The NIH stroke scale (NIHSS), Glasgow Coma Scale (GCS), symptom onset, period with abnormal yawning, blood oxygen saturation, glucose, body temperature, blood pressure, heart rate, and modified Rankin scale (mRS) were assessed for all patients. MRI lesion maps were segmented on diffusion-weighted images, spatially normalized, and the extent of overlap between the different stroke patterns was determined. Correlations between the period with abnormal yawning and the apparent diffusion coefficient (ADC) in the overlapping regions, total stroke volume, NIHSS and mRS were performed. Periods in which patients presented with episodes of abnormal yawning lasted on average for 58 h. Average GCS, NIHSS, and mRS scores were 12.6, 11.6, and 3.5, respectively. Clinical parameters were within normal limits. Ischemic brain lesions overlapped in nine out of ten patients: in seven patients in the insula and in seven in the caudate nucleus. The decrease of the ADC within the lesions correlated with the period with abnormal yawing (*r* = −0.76, Bonferroni-corrected *p* = 0.02). The stroke lesion intensity of the common overlapping regions in the insula and the caudate nucleus correlates with the period with abnormal yawning. The insula might be the long sought-after brain region for serotonin-mediated yawning.

**Electronic supplementary material:**

The online version of this article (doi:10.1007/s00429-013-0684-6) contains supplementary material, which is available to authorized users.

## Introduction

Yawning is termed abnormal or excessive if it is more frequent than generally perceived as normal, compulsive and/or not triggered by appropriate stimuli such as fatigue, boredom or contagion. At present, no definite consensus exists as to the frequency of yawns considered abnormal. The threshold of abnormality found in literature ranges from 2 yawns/10 min (Singer et al. 2007) to 30 yawns/10 min (Cattaneo et al. 2006). Abnormal yawning (or chasm) seems to be an underappreciated neurobiological phenomenon. Its cause in humans is unknown, but it can be observed in a variety of medical conditions (e.g., Thompson 2010). In contrast, physiological yawning is a ubiquitous behavioral phenomenon that can be observed across species barriers in most mammals and, according to some authors, also in most classes of vertebrates (Baenninger 1997; Guggisberg et al. 2011). A good number of clinical and pharmacological studies indicate that yawning involves the hypothalamus, particularly the paraventricular nucleus (PVN), the brainstem, and the cervical medulla (phrenic nerve C1–4 and motor supply of intercostal muscles). The neuroanatomical localization of the brainstem motor pattern that orchestrates yawning is still disputed (Askenasy 1989; Walusinski 2006). At least three distinct neural pathways have been identified that participate in the induction (and control) of yawning. These are (1) subsets of oxytocinergic neurons in the PVN that either project to the hippocampus or to the brainstem; (2) neurons in the PVN that are activated by adrenocorticotropic hormone and α-melanocyte-stimulating hormone (α-MSH), and project to the medial septum where they activate cholinergic septohippocampal neurons; (3) direct activation of septohippocampal/hippocampal neurons; and (4) a serotonergic-cholinergic pathway (e.g., to the hippocampus) whose brain localization has not been identified yet (Collins and Eguibar 2010; Sato-Suzuki et al. 1998; Argiolas and Melis 1998).

Abnormal yawning may also occur in association with cerebrovascular disease. Patients who experienced abnormal yawning with supratentorial cerebral or brainstem infarctions have been consistently reported (Singer et al. 2007; Cattaneo et al. 2006; Chang et al. 2008; Krasnianski et al. 2003; Walusinski et al. 2010). Some authors postulated a “denervation hypersensitivity” mechanism as cause of abnormal yawning. By theory, this mechanism would disconnect the putative yawning center in the brainstem from (inhibitory) control of more cranial structures, in analogy to the theories about excessive yawning in ALS patients (Williams 2000) or manifestation of enduring hiccups after medullary infarction (Park et al. 2005). Although abnormal yawning during anterior circulation (AC) stroke has been reported in the literature, investigations about the putative lesion topography and extension are still lacking. In addition, the clinical experience of abnormal yawning in cerebral ischemia has not been statistically substantiated in the literature. Here, we aimed at identifying stroke lesions in common overlapping areas of the AC that facilitate abnormal yawning. We hypothesized that the severity rather than the extension of ischemic stroke in a circumscribed strategic lesion correlates with abnormal yawning. We also aimed at identifying different neuronal pathways besides the oxytocinergic neurons in the PVN that are involved in the induction of yawning.

## Patients and methods

All ten patients were identified during an observational period of 2 years from our stroke center’s registry. Inclusion criteria were acute ischemic stroke in the AC (symptom onset <12 h), confirmed by diffusion-weighted imaging (DWI) MRI within 24 h. after stroke onset without evidence for diencephalic or brain stem lesions, and accompanied by simultaneous yawning of ≥3 times/15 min. The criteria for abnormal yawning of Singer et al. (2007) were adopted and expanded from 2 yawns/10 min to ≥3 yawns/15 min to decrease the likelihood that 2 subsequent yawns by chance were counted as one episode with abnormal yawning. Exclusion criteria were hypoxia, fever (>38 °C), and third-party anamnestic evidence for gross sleep deprivation prior to the stroke. Patients were clinically assessed at emergency entry by recording blood pressure, heart rate, body temperature, blood oxygen saturation (Biox), serum glucose levels (mmol/l), vigilance [given as Glasgow Coma Scale (GCS) score], medication, daytime of stroke onset, and the score of the National Institutes of Health Stroke Scale (NIHSS). Assessment of the Epworth Sleepiness Scale (achieved only for patient N° 7) was not feasible (little time at emergency entry, speech deficits in several patients). The modified Rankin Scale was determined at day one and between days 7–14 post-stroke. The frequency of abnormal yawning was monitored at random hours several times a day by the emergency neurologist (for the most part HK). Abnormal yawning was defined as suspended, if patients yawned <3 times/15 min on two separate clinical visits during the same day. Thus, the period with abnormal yawning was defined as the time (in hours) between stroke onset and 9 p.m. on the day before bouts of abnormal yawning could not be observed anymore. 9 p.m. was chosen because it is the average bedtime for patients in our hospital. All values are given as mean ± SD. The observational study was approved by the local ethics committee.

### Lesion analysis

MRI scans included transverse DWI, which was performed at 1.5T using a 2D echo-planar imaging sequence with an 256 × 256 image matrix with 0.94 mm pixel resolution and 100 % sampling in all directions, slice thickness 5 mm, slice spacing 1.5 mm, echo time 137 ms, and three-directional orthogonal diffusion gradients with three b-values of *b* = 50, 500 and 1,000 s/mm^2^, from which the ADC maps were obtained. Brain areas, encompassing restricted diffusion after acute ischemic stroke, were identified by a board-certified neuroradiologist (CW) and manually traced in native space on DWI scans using MRIcroN (http://www.mccauslandcenter.sc.edu/mricro/mricron/index.html/), yielding binary lesion maps. DWI maps were co-registered to the EPI-template using SPM5 (http://www.fil.ion.ucl.ac.uk/spm/software/spm5/). The lesion maps and DWI images were spatially normalized to Montreal Neurological Institute (MNI) stereotaxic space using the unified segmentation algorithm in SPM5. For inter-individual comparisons, images of patients with left-sided lesions were flipped to the contralateral hemisphere. Group-specific lesion overlay plots were created using MRIcroN.

### DWI restrictions and ADC analysis

Several studies demonstrated that decreased apparent diffusion coefficient (ADC) values are inversely correlated with stroke outcome, and that within DWI lesions the severity of neuronal injury reflects the degree of ADC alteration (Engelter et al. 2003; Schwamm et al. 1998). Besides total stroke volumes, we thus measured the ADC, a marker for the intensity of ischemic lesions, in the commonly involved overlapping regions of the caudate nucleus and the insula for correlation analysis with the period with abnormal yawning.

### Statistics

We used the time period in which patients presented with episodes of abnormal yawning as a measure of its clinical impact. We calculated the non-parametric Spearman correlation coefficient between the period length and four parameters of interest, two of which are clinical scores (NIHSS for stroke severity and mRS for outcome), and two of which are imaging parameters (total stroke volume and ADC for stroke severity). Because the involvement of only one of the two identified regions (caudate and insula) was sufficient for abnormal yawning, the ADC of the more severely involved of these two regions (the lower of the two ADC values) was used for correlation analysis. Significance for the obtained correlation coefficient *r* was calculated as *p* value and Bonferroni-corrected for multiple comparisons (for the above four independent correlation tests).

## Results

The aim of the study concerns the identification of common overlapping lesions in patients with abnormal yawning and the correlation of their stroke severity with the period with abnormal yawning. Clinical findings and imaging characteristics are summarized in Table 1. In none of the 10 patients, abnormal yawning was accompanied by pandiculations (stretching of trunk and extremities) or by parakinesia brachialis oscitans, a phenomenon whereby patients move their paralyzed extremities while yawning (Walusinski et al. 2010). Symptom onset occurred during daytime in seven patients. Patient N^o^ 3 had a wake-up stroke. Patient N^o^ 1 fulfilled the clinical and imaging criteria of early subacute infarction (>6 h, increased signal on T2w images and being for the last time clinically asymptomatic <16 h ahead of admission). On admission with abnormal yawning, all 10 patients were normoxic (>92 % blood oxygen), afebrile (mean body temperature 36.5 ± 0.5 °C), and serum glucose levels were 6.4 ± 1.1 mmol/l (normal range 3.33–5.55). Average blood pressure was 139 ± 28/75 ± 14 mmHg, average heart rate (74 ± 23 bpm), and average NIHSS and GCS were 11.6 ± 6.8 and 12.6 ± 2.5, respectively. Thus, there was no evidence for other potential causes of frequent yawning such as hypoxia, low glucose levels, increased body temperature, time of the day with higher yawning incidence due to circadian rhythm (70 % of strokes occurred during daytime), or increased sleepiness/decreased vigilance (average GCS was 12.6). Potential sedating side effects of medication could not be excluded as additional cause for drowsiness and higher yawning incidence in individual patients. However, no clear association between intake of frequently sedating medication and decreased vigilance (GCS) was seen (Supplementary Table 1). It was therefore concluded that abnormal yawning likely arose from the ischemic brain lesions. The mRS was 3.5 ± 1.5 at day one. The second mRS was determined between days 7–14 post-stroke and its average score was 3.0 ± 1.6 (Table 1). Patients were daily visited and amongst other things observed for increased yawning frequency. During follow-up, the period with abnormal yawning lasted for an average 58 ± 24 h.

**Table 1:** Descriptive data of stroke patients

Patient No.	Age (years)/sex	Neurological symptoms	NIHSS	DWI-MRI findings	Stroke symptom onset (daytime)	Period length with abnormal yawns (hours)	Biox (%)	Glucose (mmol/l)	Vigilance (GCS)	Temperature (°C)	Systol./diastol. BP (mmHg)	Heart rate (bpm)	mRS d1	mRS d7–14
1	90/f	Global aphasia, deviation conjugée left, brachio-facial paresis right	6, deteriorating	Posterior third of left MCA territory	6:00–11:30	40.0	100 with 3 l O2	5.2	11	36.5	175/95	135	4	3
2	74/f	Multimodal neglect to the left, facial paresis left, disorientation	4	Insula, caput nucleus caudate right	20:00	25.0	100 with 3 l O2	6.9	14	36.5	180/60	62	1	1
3	56/m	Global aphasia, right-sided senso- motor hemisyndrome	12	Fronto-opercular left including external capsule, dorsal putamen	22:00–1:45	43.2	93	7.2	11	35.5	137/80	76	3	2
4	62/m	Dysarthria, right-sided facial and distal arm paresis	6, deteriorating	Multiple small DWI lesions in precentral gyrus and parieto-occipital left	5:45	63.2	100	8.2	15	37.6	135/79	68	5	5
5	68/m	Global aphasia, hemiplegia right	15	Anterior 2/3 of MCA territory	15:40	77.3	96	7.4	12	36.4	115/70	74	5	5
6	71/m	Global aphasia, right-sided senso-motor hemisyndrome	18	Left frontal and temporal opercular region, insula	11:00	96.0	94	6.8	9	36.8	160/90	60	5	5
7	74/m	Dysarthria, left-sided senso-motor hemisyndrome	5	Cortical area of right pre-/postcentral gyrus	17:30	27.5	95	5.0	15	36.0	150/70	60	3	2
8	79/m	Dysarthria, left-sided facial and arm paresis	3	Right temporal operculum, posterior insula, basal ganglia	12:00	57.0	96	6.5	15	36.7	90/60	80	2	1
9	89/m	Deviation conjugée to the left, global aphasia, senso-motor hemiparesis right	21	Left basal ganglia	19:10	73.8	96	5.0	9	36.7	134/91	64	5	4
10	65/m	Dysarthria, senso-motor hemisyndrome left, neglect to the left	15	Anterior insula	12:45	80.2	94	5.6	15	36.6	120/59	60	2	2
Average			11.6			58.3		6.4	12.6	36.5	139/75	73.9	3.5	3.0
SD			6.8			23.9		1.1	2.5	0.5	28/14	22.7	1.5	1.6

Neurological deficits and DWI restrictions at their time of emergency admission: listed are ratings on the NIHSS stroke scale (NIHSS), the Glasgow Coma Scale (GCS), and modified Rankin Scale determined on day one (d1) and between days 7–14 (d7–14) post-stroke, symptom onset in hours and minutes, period length with abnormal yawning in hours, blood oxygen saturation in % (Biox), serum glucose levels, body temperature, systolic and diastolic blood pressure (BP), and heart rate in beats per minute (bpm). All data are provided as average ± standard deviation (SD)

**Fig. 1:**
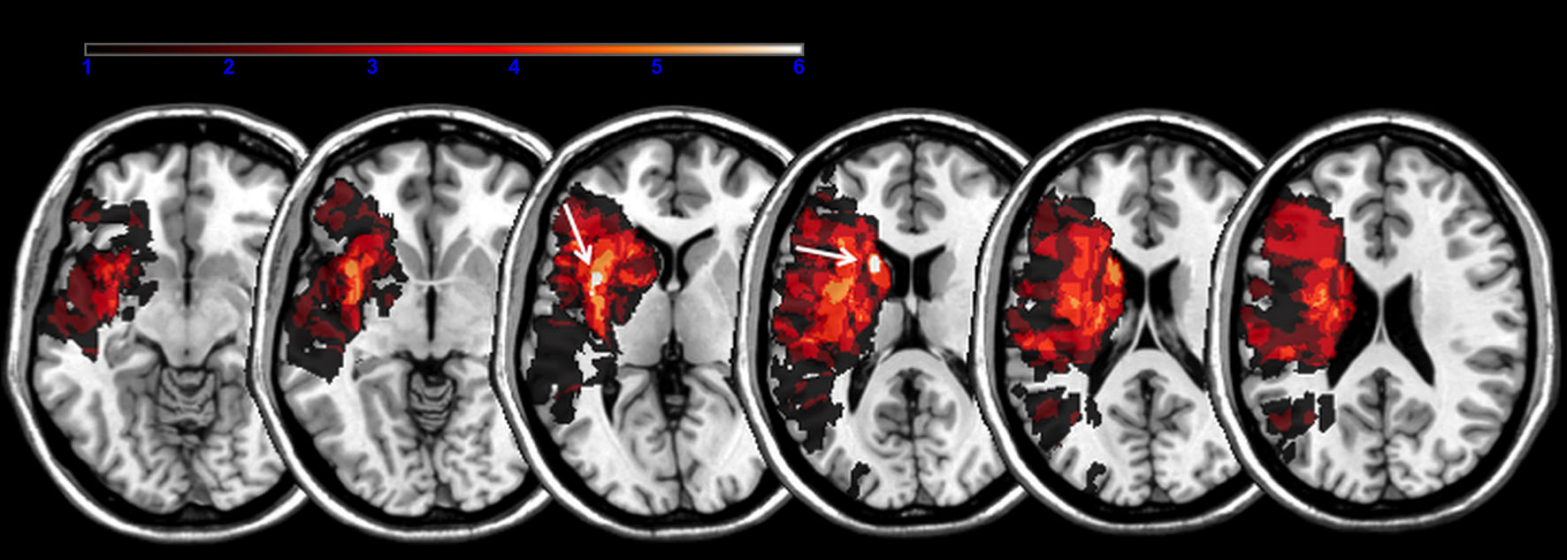
Lesion maps. Lesion overlay maps incorporating seven patients with common lesions in the insula (*arrow* MNI: *x* = −37, *y* = 7, *z* = 5) and seven in the caudate head (*arrow* MNI: *x* = −17, *y* = 14, *z* = 15) associated with abnormal yawning

Six patients had right-hemispheric and four patients left-hemispheric strokes. The lesion map extension in the cohort included 34,073 lesioned voxels (313.8 cm^3^) and overlapped within 365 voxels (0.36 cm^3^; maximum overlap at MNI: *x* = −37, *y* = 7, *z* = 5) in 7 patients in the insula, and within 265 voxels (0.27 cm^3^; maximum overlap at MNI: *x* = −17, *y* = 14, *z* = 15) in seven patients in the caudate head (Fig. 1). Patients N° 1, 2, 4, 5 and 9 had DWI restrictions both in the caudate head and insula; patients N° 3 and 6 in the insula only; N° 8 and 10 in the caudate head only.

**Fig. 2:**
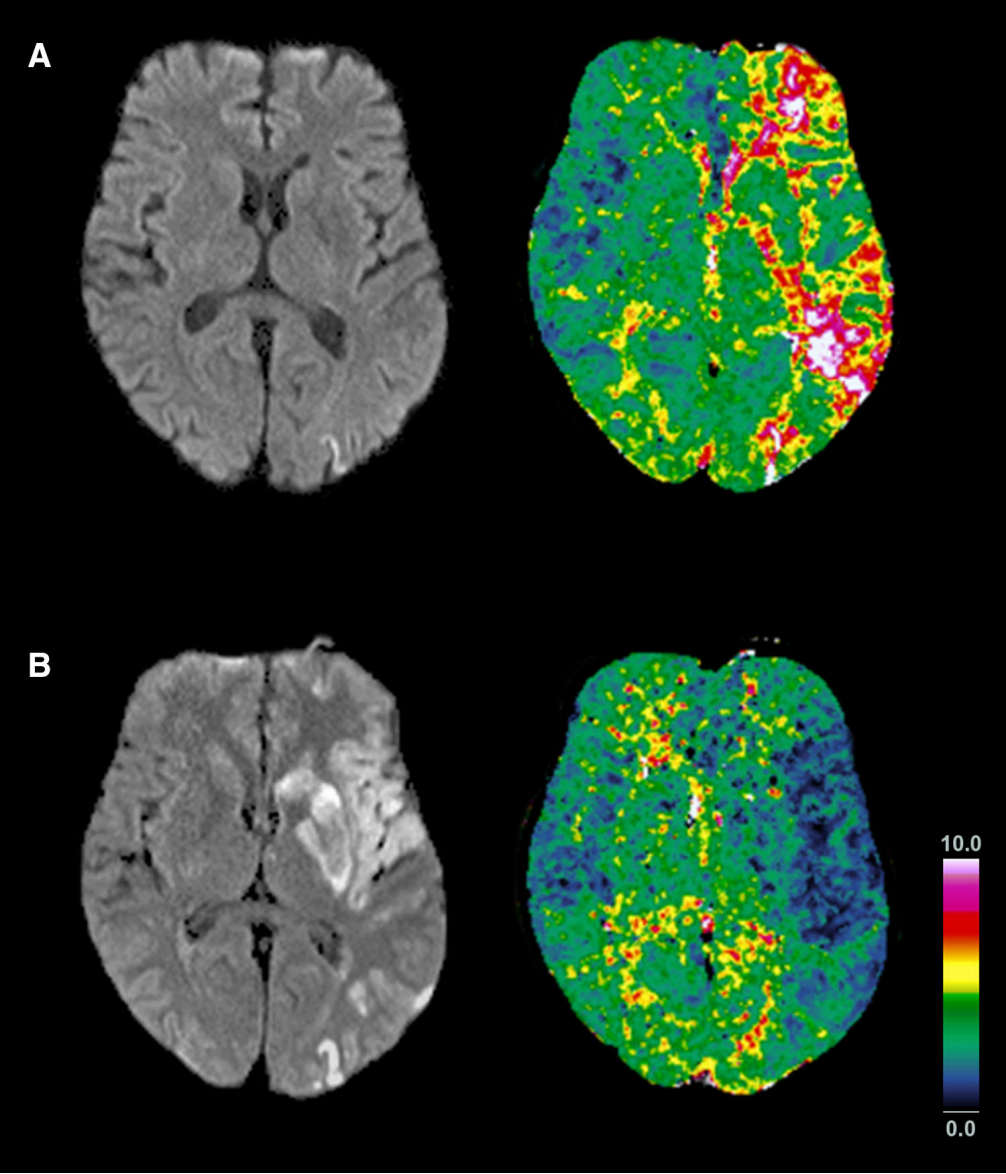
Abnormal yawning without initial DWI restrictions. (**a**) Evolution of the penumbra in a patient with abnormal yawning initially not related to DWI lesions in the caudate head or insula (Pat N^o^ 4). While cortical DWI restrictions were initially restricted to the frontal lobe (not shown) and parietal lobe, perfusion imaging revealed a widespread penumbra along the left MCA encompassing the insula and caudate head (TTP delay >4.5 s). (**b**) Follow-up after 48 h revealed prolonged infarction of the tissue at risk in the left insula, striatum and frontal and parietal lobe, now including the caudate head and the insula, with luxury perfusion of the infarcted tissue

Patient N° 4 was exceptional because at admission, he presented with abnormal yawning, slight dysarthria, and mild brachio-facial hemiparesis, while his MRI revealed a penumbral impairment of perfusion with a time to peak (TTP) delay >4.5 s encompassing the posterior insula (Fig. 2a), consistent with lesioned function of this region, but initially without DWI restrictions and thus potentially reversible. Subsequently, he clinically deteriorated and, in keeping with the increased TTP delay, a subsequent infarction manifested including the insula and caudate head after 48 h. (Fig. 2b). Patient N° 7 presented with ischemic lesions in the postcentral gyrus, angular gyrus and superior temporal gyrus adjacent to but sparing the insula and caudate head, and only had a short period with abnormal yawning.

**Fig. 3:**
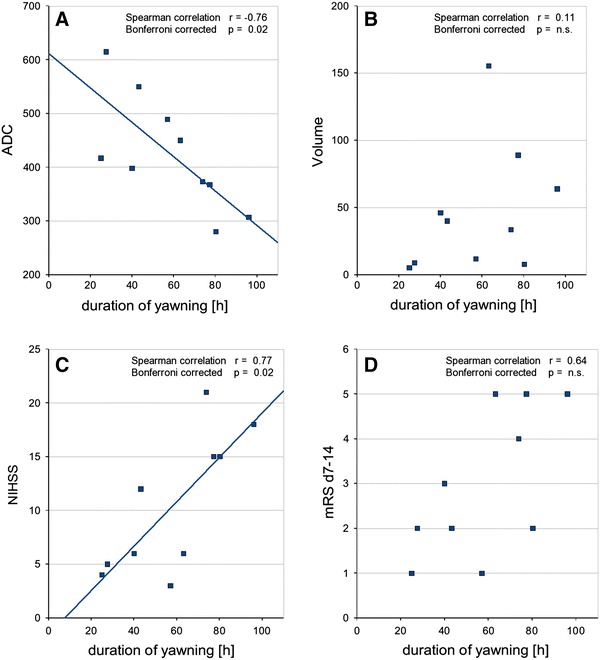
Abnormal yawning and clinical and neuroradiological stroke parameters. Correlations of the period length with abnormal yawning (duration of yawning [*h*]) with: (**a**) ADC values as neuroradiological surrogate marker of stroke severity, and (**c**) NIHSS as clinical marker of stroke severity. No correlations were found between duration of yawning and (**b**) neuroradiological stroke volume, and (**d**) mRS d7–14 as clinical outcome marker, determined between days 7–14 after stroke onset

The period of abnormal yawning correlated negatively with the ADC (*r* = −0.76, Bonferroni-corrected *p* = 0.02, raw *p* = 0.006) and positively with the NIHSS (*r* = 0.77, Bonferroni-corrected *p* = 0.02, raw *p* = 0.005), but not with total stroke volume (*r* = 0.11, Bonferroni-corrected *p* = n.s) nor with mRS (*r* = 0.64, Bonferroni-corrected *p* = n.s) (Fig. 3a–d).

Follow-up imaging was analyzed to investigate whether the brain areas with the strongest ADC decrease resulted in permanent tissue destruction (Table 2).

**Table 2:** Follow-up imaging of stroke patients

Patient No.	Post-stroke follow-up	Time to follow-up	Topology	Stroke extension
1	None			
2	CT	24 h	NC+, Ins+	MCA M1 occlusion^a^
3	MRI	24, 120 h	Ins+	MCA M2 occlusion^a^
4	MRI	48 h	NC+, Ins+	MCA M1 occlusion^a^
5	CT	24 h	NC+, Ins+	MCA M1 occlusion^b^
6	CT	24, 120 h	Ins+	MCA M1 occlusion^b^
7	None			
8	MRI	Chronic	Ins+	MCA M1 occlusion
9	CT	24, 72 h	NC+, Ins+	MCA M1 occlusion^a^
10	CT	24 h	NC+, Ins+	MCA M1 occlusion^a^

Same patient numbering as in Table 1. Two patients received no follow up examination due to early transfer to other hospitals. The remaining eight patients developed permanent tissue damage at the corresponding areas on follow-up imaging. “Chronic” in the column “time to follow-up” denotes cerebral imaging ≥1 year after stroke. The following abbreviations are used: NC+, permanent tissue damage (CT-hypodense, MRI T2/FLAIR hyperintense) in caudate nucleus; Ins+, permanent tissue damage (CT-hypodense, MRI T2/FLAIR hyperintense) in insula; *MCA*, middle cerebral artery; M1, M1 segment of MCA; M2, M2 segment of MCA

^a^i.a. Thrombolysis, mechanical recanalisation

^b^i.v. Thrombolysis

## Discussion

In this observational study, we investigated the spatial topography of stroke lesions linked to abnormal yawning. We present the first statistically substantiated evidence that ischemic lesions in the posterior insula and caudate nucleus facilitate abnormal yawning. Within these two regions, the ischemia intensity—as measured by the extent of the ADC signal drop—correlated with the period of abnormal yawning after stroke onset. Significant correlations were further observed between the period of abnormal yawning and clinical stroke severity as measured by NIHSS, and a trend for correlation with mRS that did not pass significance correcting for multiple testing. Total stroke volume did not correlate at all with the duration of yawning, further supporting the specificity of the two identified small regions of overlap as opposed to the extent of the whole stroke.

### Abnormal yawning in anterior circulation stroke

A previous study by Singer et al. (2007) identified supratentorial lesions in patients with extended AC strokes and implicated “that excessive yawning can be a sign of supratentorial lesions affecting the MCA (medial cerebral artery, comment ours) territory”. They hypothesized that supratentorial lesions may release the hypothalamic PVN from neocortical control mechanisms along the hippocampus and periamygdalar region, thereby increasing its activity and leading to abnormal yawning. The authors identified no ischemic diencephalic lesions in their CT study, however, exclusion of additional affections of the brainstem failed due to the methodological limitations of CT technology (Chalela et al. 2007; Masdeu et al. 2006). The current MR-study adds further evidence to the hypothesis that ischemic lesions of the AC are related to abnormal yawning without evidence of brainstem lesions. We also ruled out lesions of the PVN, thereby supporting the hypothesis that ischemic lesions of the AC cause disinhibition of subcortical nuclei or networks that control yawning (Singer et al. 2007). In contrast, the extension of ischemic areas to more than one-third of the MCA territory, as previously suggested (Singer et al. 2007) could not be confirmed as being causative for abnormal yawning in our study. Our patients were less severely affected by MCA stroke extension according to the DWI stroke extent, the NIHSS, and mRS scores than previously reported (Singer et al. 2007). Our positive correlation between clinical stroke severity (NIHSS) and the period with abnormal yawning may merely correspond to an epiphenomenon representing the higher probability of strokes involving deep brain areas such as the basal ganglia/caudate manifesting with severe deficits, as opposed to strokes confined to more peripheral regions of the brain.

### Abnormal yawning due to caudate nucleus lesions

Abnormal yawning was observed in case reports with isolated caudate lesions by Renau-Lagranja et al. (2010; stroke in caudate) and Auer et al. (1987; cysticercosis lesion in caudate), and is supported by our data. As hypothesis, we ascribe this clinico-anatomical association to an excess release of dopamine and acetylcholine due to ischemic damage in the caudate nucleus. The striatum, including the caudate nucleus, encompasses a high density of dopaminergic and cholinergic terminals. The caudate nucleus is highly innervated by dopamine neurons that originate mainly from the ventral tegmental area and the substantia nigra pars compacta. Animal studies demonstrated a release of dopamine and glutamate neurotransmitter levels during ischemic stroke (Richards et al. 1993). Furthermore, animal experiments demonstrated the dependence of yawning frequency on dopaminergic neurotransmission by an activation of D2/D3 receptors (Baladi et al. 2010; Depoortère et al. 2009). The negative correlation between decreased ADC in the caudate nucleus and prolonged periods with abnormal yawning is thus in favor of a disruption of dopaminergic projections with subsequent uncoordinated release of dopamine levels that may facilitate abnormal yawning.

### Abnormal yawning due to insular lesions

The insula is not known to be a direct target of the mesotelencephalic dopamine system. It is intensively connected with other cortical and subcortical regions via a cortico-striato-thalamic network (linking the insula also to the caudate nucleus; see Metzger et al. 2010), with the lateral hypothalamus (Jasmin et al. 2004), the hippocampus [at least with the entorhinal cortex (Mesulam and Mufson 1982)], and the brainstem via corticobulbar pathways (Jasmin et al. 2004; Ruggiero et al. 1987). We refer for further review of the neuroanatomy and function of the insula to the classical work by Mesulam and Mufson (1985) and an excellent new work by Nieuwenhuys (2012). Monosynaptic trajectories from the posterior insula or the caudate nucleus head are not known to directly project to the hypothalamic PVN. Therefore, our data support the involvement of additional pathways/mechanisms in control of the yawning motor pattern. In line with our hypothesis that abnormal yawning is mainly caused by a denervation hypersensitivity mechanism or excessive neurotransmitter release (or both) due to targeted and intensive disruption of core areas within the AC, we envisage three scenarios. First, a (GABAergic?) disinhibition of insular targets such as the entorhinal cortex, lateral hypothalamus or the brainstem might lead to abnormal yawning. Interestingly, mono-/oligosynaptic projections from the posterior insula to the Raphe nucleus and the nucleus tractus solitarius (NTS) exist (Allen et al. 1991; Saper 1982, 2000). As the NTS is located in the vicinity of the Pre-Bötzinger complex (a neuronal respiratory rhythm generator in the ventrolateral medulla) and the cranial nerve nuclei V, VII, IX, X and XII [which are involved in yawning; see Smith et al. (1991); Abdala et al. (2009)], it is conceivable that ischemic lesions in the posterior insula may not only affect swallowing, taste and cardiovascular events (Cereda et al. 2002; Brandt et al. 1995), but also the frequency of yawning. Second, the pharmacology of the insula contains a series of neurotransmitters and receptors (albeit with sometimes indirect evidence), including GABA, glutamate, acetylcholine and serotonin (Jasmin et al. 2004; Chen et al. 2010; Van De Werd et al. 2010; Tuerke et al. 2012). Interestingly, infusion of serotonin agonists into the insula induced gaping in awake rats, which was interpreted as conditioned disgust (Tuerke et al. 2012), but can also be part of the yawning event without stretching. It is tempting to speculate that ischemia of variable severity with only partially destructive neuronal lesions in the insula leads to excessive serotonin release and induction of yawning. Serotonin-mediated yawning is known to occur independently of the PVN. The brain regions responsible for serotonin-mediated yawning had not been identified yet. Third, the actually responsible regions may not be the insula itself, but the adjacent white matter tracts (capsula extrema) or the claustrum, which may not necessarily be reliably separated from the insular cortex with the resolution of the spatial renormalization techniques, which were needed to identify inter-individual overlaps, and because the underlying regions are likely to be involved to a similar degree due to the common vascular supply including common collateralization pathways.

Finally, it was previously stated (Walusinski 2006) that another function of yawning may be to check for the homeostasis of inner organs and perceive a feeling of wellbeing, based on the observations that visceral afferents arrive in posterior insular cortex while processing of self-awareness takes place in the anterior insula (von Economo neurons), and because the insula may be (indirectly) activated in the yawning process. We have not systematically analyzed our patients for their retained or lost ability to check for their wellbeing of inner organs, but this idea may be explored in future studies.

### Abnormal yawning due to other etiologies

Our own study only assessed yawning in ischemic stroke. The literature also mentions a few cases of yawning associated with stroke in the insula or the caudate nucleus. Other (non-stroke) etiologies for yawning in the literature involve traumatic brain injury (Laurent-Vannier et al. 1999), brain surgery (Martino et al. 2012), and complex focal seizures (Penfield and Jasper 1954). However, we have failed to identify a study that reported hyperammonemia inducing abnormal yawning.

### Limitations of the study

The structural analysis revealed DWI restrictions in the insula and caudate nucleus, but these findings did not appear as a prominent feature in every patient. Nevertheless, the correlation demonstrates that patients with less pronounced involvement of these regions also show a shorter duration of yawning. Moreover, effects of restricted perfusion in the penumbra in patient N^o^ 4 without initial DWI restrictions in the core regions, as well as DWI restrictions near the posterior insula as in patient N^o^ 7, may have caused a functional disruption of cortico-subcortical pathways comparable to ischemic stroke in these regions. Since lesions in the core regions (the insula and the caudate head) are frequently detected in patients without abnormal yawning, further studies are required to better specify the causative lesions with higher power and improved techniques such as tractography and structural connectivity measures. Future studies also have to systematically capture the transmitters used by insular trajectories.

## Conclusions

We provide the first statistically substantiated study that ischemic stroke in two specific regions within the AC can indeed be associated with abnormal yawning in few cases. We add to the existing evidence that (a) strokes do not necessarily have to be severe (high NIHSS) to elicit abnormal yawning; (b) in general, the intensity but not the extent of ischemia in core regions within the AC correlates with duration of abnormal yawning; (c) additional pathways and/or mechanisms besides the hypothalamus may be involved in abnormal yawning. The as yet unknown brain region of serotonin-mediated yawning might be the insula.

The hypothesis of excessive uncoordinated neurotransmitter release due to ischemic lesions might be one explanation why the other neurological stroke deficits caused by the same stroke frequently outlast abnormal yawning. This transient nature of abnormal yawning might also be the explanation why it is perceived as rare phenomenon in acute neurological disorders.

## Electronic supplementary material

Below is the link to the electronic supplementary material.

Supplementary material 1 (PDF 41 kb)

Supplementary Table 1: potentially sedating side effects of medication and vigilance according to GCS. Identical patient numbering as in Table 1. GCS and medication at emergency entry are listed for each patient. Medication with potentially sedating side effects is highlighted in bold, followed by frequency of observed sleepiness in brackets according to Swissmedic(http://www.swissmedicinfo.ch). Occ, occasionally: < 1 % & ≥ 0.1 %; freq, frequent: < 10 % & ≥ 1 %; very freq, very frequent: ≥ 10 %. Note that patients N° 8, 10 took medication which may frequently lead to sleepiness but had full vigilance. In contrast, decreased vigilance could be seen in patients who took no medication or medication that only occasionally leads to sleepiness (e.g., patients N° 3, 6).
