# Supplementary material for: Insular and caudate lesions release abnormal yawning in stroke patients
Source: Brain Struct Funct. 2013 Dec 12;220(2):803–12. doi: 10.1007/s00429-013-0684-6 (PMC4341028; doi:10.1007/s00429-013-0684-6)
Supplement: Supplementary file 1 — Supplementary material 1 (PDF 41 kb) [file 429_2013_684_MOESM1_ESM.pdf]

Supplementary Table 1

Patient No	Vigilance (GCS)	medication
1.	11	phenprocoumon, perindopril, **verapamil** (occ), acetylcystein, **esomeprazol** (occ), levothyroxin, mebeverin, **trimipramin** (freq)
2.	14	**atenolol** (freq)
3.	11	none
4.	15	none
5.	12	aspirin, clopidogrel, **bisoprolol** (freq), perindopril, metformin and glibenclamid, pravastatin, **nicorandil** (freq), **omeprazol** (occ)
6.	9	**enalapril** (occ), **carvedilol** (occ), **felodipin** (occ)
7.	15	clopidogrel
8.	15	phenprocoumon, **bisoprolol** (freq), atorvastatin
9.	9	phenprocoumon, **metoprolol** (very freq), **enalapril** (occ), isosorbide dinitrate, **nicorandil** (freq)
10.	15	**methyl phenidate** (occ), **sertraline** (freq)
